# Supplementary material for: Quantitative Risk Evaluation of Antimicrobial-Resistant Vibrio parahaemolyticus Isolated from Farmed Grey Mullets in Singapore
Source: Pathogens. 2023 Jan 5;12(1):93. doi: 10.3390/pathogens12010093 (PMC9867465; doi:10.3390/pathogens12010093)
Supplement: Supplementary file 1 [file pathogens-12-00093-s001.zip › pathogens-2108238-supplementary.pdf]

Supplementary Information

Table S1 – Confirmation of antimicrobial resistance of *Vibrio parahemolyticus* bacterial isolates via disc diffusion method.

	Treatment		
	Ampicillin	Penicillin G	Tetracycline
Total bacterial isolates counts	27	14	5
Total resistant isolates counts (%)	25 (92.6)	14 (100)	4 (80)

Table S2 – Antimicrobial susceptibility profile of *V. parahaemolyticus* isolates via disc diffusion method.

Antibiotics	Interpretive results (farm)			Interpretive results (retail)		
	Sensitive (%)	Intermediate (%)	Resistant (%)	Sensitive (%)	Intermediate (%)	Resistant (%)
Ampicillin (10 µg)	4 (9.80)	10 (24.4)	27 (65.8)	2 (3.17)	8 (12.7)	53 (84.1)
Ampicillin/ sulbactam (20 µg)	35 (85.4)	-	6 (14.6)	62 (98.4)	-	1 (1.60)
Pencillin G (10 unit)	-	-	41 (100)	-	1 (1.60)	62 (98.4)
Tetracycline (30 µg)	34 (82.9)	7 (17.1)	-	58 (92.1)	1 (1.60)	4 (6.30)
Cefotaxime (30 µg)	34 (82.9)	7 (17.1)	-	57 (90.5)	6 (9.50)	-
Ciprofloxacin (5 µg)	31 (75.6)	10 (24.4)	-	57 (90.5)	6 (9.50)	-
Sulfamethoxazole/ Trimethoprim (25 µg)	41 (100)	-	-	63 (100)	-	-
Chloramphenicol (30 µg)	41 (100)	-	-	63 (100)	-	-

Table S3 – Standard errors of occurrence and concentration data of haemolytic *V. parahaemolyticus* within the farm-to-home and retail-to-home chain scenarios.

			Farm-to-home				Retail-to-home			
			Unsupplemented	Ampicillin	Penicillin G	Tetracycline	Unsupplemented	Ampicillin	Penicillin G	Tetracycline
	Occurrence (Farm/Retail)		5.46E-05	1.98E-05	2.32E-05	N.A.	5.44E-05	5.14E-05	2.99E-05	6.45E-05
Concentration (LogCFU/g)	Farm	Pre-harvest	6.92E-04	4.52E-04	2.00E-04	N.A.	N.A.	N.A.	N.A.	N.A.
		Post-harvest	6.62E-04	4.74E-04	2.20E-04	N.A.	N.A.	N.A.	N.A.	N.A.
	Retail	Retail-start	6.62E-04	4.73E-04	2.23E-04	N.A.	4.19E-04	5.68E-04	6.60E-04	6.78E-04
		Retail-end	6.68E-04	4.79E-04	2.29E-04	N.A.	4.28E-04	5.66E-04	6.65E-04	6.76E-04
	Home	Home	6.69E-04	4.78E-04	2.18E-04	N.A.	4.39E-04	5.72E-04	6.79E-04	6.66E-04
	Preparation	Average Washing	7.02E-04	2.36E-04	1.27E-04	N.A.	5.51E-04	6.27E-04	7.08E-04	5.53E-04
		Washing	6.67E-04	1.73E-04	3.79E-05	N.A.	4.82E-04	5.96E-04	6.46E-04	4.54E-04
		No washing	7.55E-04	5.13E-04	2.45E-04	N.A.	4.91E-04	6.78E-04	7.14E-04	6.37E-04
	Cooking	Average Washing	2.60E-04	2.70E-05	2.47E-06	N.A.	1.38E-04	1.51E-04	2.61E-04	1.18E-04
		Washing	2.39E-04	1.20E-05	1.33E-07	N.A.	1.21E-04	1.53E-04	2.06E-04	8.87E-05
		No washing	4.87E-04	4.20E-05	4.16E-06	N.A.	2.65E-04	3.24E-04	3.81E-04	2.21E-04

Table S4 – Standard errors of risk estimates data across all scenarios.

		Farm to home			Retail to home		
		$P_{ill,serving}$	$P_{ill,yearly}$	N_{cases}	$P_{ill,serving}$	$P_{ill,yearly}$	N_{cases}
Unsupplemented	Average Washing	5.28E-06	5.98E-05	5.39E-01	8.36E-07	3.62E-05	3.27E-01
	Washing	4.19E-06	5.76E-05	5.19E-01	6.89E-07	3.26E-05	2.94E-01
	No washing	1.13E-05	1.18E-04	1.06E-00	3.83E-06	8.63E-05	7.78E-01
Ampicillin	Average Washing	2.87E-08	6.04E-06	5.44E-02	1.03E-06	3.37E-05	(3.03E-01
	Washing	6.04E-09	2.60E-06	2.34E-02	7.17E-07	3.51E-05	3.16E-01
	No washing	8.38E-08	1.07E-05	9.65E-02	3.66E-06	7.75E-05	6.99E-01
Penicillin G	Average Washing	8.89E-10	6.02E-07	5.43E-03	2.98E-06	6.63E-05	5.98E-01
	Washing	4.14E-11	3.25E-08	2.93E-04	1.76E-06	4.58E-05	4.13E-01
	No washing	1.79E-09	1.37E-06	1.24E-02	7.83E-06	1.12E-04	1.01
Tetracycline	Average Washing	N.A.	N.A.	N.A.	7.80E-07	2.05E-05	1.85E-01
	Washing	N.A.	N.A.	N.A.	4.14E-07	2.07E-05	1.87E-01
	No washing	N.A.	N.A.	N.A.	2.53E-06	6.29E-05	5.67E-01
